# Marked Ethnic, Nativity, and Socioeconomic Disparities in Disability and Health Insurance among US Children and Adults: The 2008–2010 American Community Survey

**DOI:** 10.1155/2013/627412

**Published:** 2013-10-22

**Authors:** Gopal K. Singh, Sue C. Lin

**Affiliations:** ^1^US Department of Health and Human Services, Health Resources and Services Administration, Maternal and Child Health Bureau, 5600 Fishers Lane, Room 18-41, Rockville, MD 20857, USA; ^2^US Department of Health and Human Services, Health Resources and Services Administration, Bureau of Primary Health Care, 5600 Fishers Lane, Room 6A-55, Rockville, MD 20857, USA

## Abstract

We used the 2008–2010 American Community Survey Micro-data Sample (*N* = 9,093,077) to estimate disability and health insurance rates for children and adults in detailed racial/ethnic, immigrant, and socioeconomic groups in the USA. Prevalence and adjusted odds derived from logistic regression were used to examine social inequalities. Disability rates varied from 1.4% for Japanese children to 6.8% for Puerto Rican children. Prevalence of disability in adults ranged from 5.6% for Asian Indians to 22.0% among American Indians/Alaska Natives. More than 17% of Korean, Mexican, and American Indian children lacked health insurance, compared with 4.1% of Japanese and 5.9% of white children. Among adults, Mexicans (43.6%), Central/South Americans (41.4%), American Indians/Alaska Natives (32.7%), and Pakistanis (29.3%) had the highest health-uninsurance rates. Ethnic nativity disparities were considerable, with 58.3% of all Mexican immigrants and 34.0% of Mexican immigrants with disabilities being uninsured. Socioeconomic gradients were marked, with poor children and adults having 3–6 times higher odds of disability and uninsurance than their affluent counterparts. Socioeconomic differences accounted for 24.4% and 60.2% of racial/ethnic variations in child health insurance and disability and 75.1% and 89.7% of ethnic inequality in adult health insurance and disability, respectively. Health policy programs urgently need to tackle these profound social disparities in disability and healthcare access.

## 1. Introduction

The racial/ethnic composition of the US population has undergone substantial change in recent decades [1, 2]. The proportion of the White population in the US declined from 87.6% in 1970 to 63.3% in 2011, whereas the percentage of Black population increased slightly from 11.1% to 12.2% during the same time period [1, 2]. On the other hand, the Hispanic population increased rapidly from 9.1 million (4.5%) in 1970 to 51.9 million (16.7%) in 2011, whereas the Asian/Pacific Islander population increased nearly 5-fold, from 3.7 million (1.6%) in 1980 to 18.2 million (5.8%) in 2011 [1–5]. Changes in the racial/ethnic composition have occurred primarily as a result of large-scale immigration from Latin America and Asia during the past four decades [6–9]. The immigrant population grew from 9.6 million in 1970 to 40.4 million in 2011 [2, 6–9]. Immigrants currently represent 13.0% of the total US population [2]. Over 80% of all US immigrants currently hail from Latin America and Asia, in contrast to 1960 when Europeans accounted for 75% of the foreign-born population [6–8]. Increase in the number of immigrant children has also been substantial, with the number doubling from 8.2 million in 1990 to 17.5 million in 2011 [2, 10]. In 2011, nearly a quarter of US children had at least one foreign-born parent [2, 10]. 

Despite such marked increases in the immigrant population and growing ethnic heterogeneity of the US population, analysis of health inequalities according to detailed ethnic and national origins, particularly among recent ethnic and immigrant groups from Asia such as those from the Indian subcontinent, Korea, Vietnam, Laos, Cambodia, and Thailand, remains relatively uncommon [11–17]. Besides the 2000 and prior decennial censuses, the American Community Survey (ACS) is the only contemporary national data source in the USA that provides extensive socioeconomic, demographic, disability, and health insurance information for a large number of ethnic groups and countries of origin, including some of the newly arrived ethnic groups from Asia, Africa, Latin America, and the Caribbean [1, 2, 6, 13–20]. 

Disability is a major morbidity and health status indicator both in the United States and globally [21–24]. More than a billion people, about 15% of the world's population, are estimated to have some form of disability [21]. Disability rates have been rising in many countries of the world due to population aging and increases in chronic health conditions [21]. In 2011, an estimated 37.2 million people (12%) in the US had disability [2]. In the USA and across the world, people with disabilities are more likely to report poorer physical and mental health status, higher rates of smoking, physical inactivity, obesity, and alcohol use, lower income and educational achievements, higher poverty and unemployment rates, and experiencing more barriers in accessing social, economic, transport, and healthcare services than people without disabilities [2, 21, 23, 25]. Health insurance coverage is a major determinant of access to healthcare [22]. Although in much of the industrialized world, healthcare coverage is generally available to all citizens, 46.4 million Americans, including 5.5 million US children, were without health insurance in 2011 [2, 26]. Research has shown that uninsured individuals are much more likely to delay or forego preventive health services and needed medical care, have higher rates of mortality, and are more likely to be diagnosed with an advanced stage disease than individuals with health insurance [22, 26–28]. 

Although previous research has examined racial/ethnic and nativity disparities in disability rates in the USA using the 1990 and 2000 decennial censuses, disability and health uninsurance rates have not been analyzed for both children and adults from detailed ethnic and immigrant groups [13–17]. Although substantial ethnic, nativity, and socioeconomic inequalities in health, life expectancy, all-cause and cause-specific mortality, and chronic disease conditions are well documented, such inequalities in disability have been less well studied [11–17, 22, 29–32]. Analyzing social inequalities in disability is important because ethnic and socioeconomic characteristics can significantly influence factors underlying the disablement process, including the development of physical and mental impairments, comorbidities, health-risk behaviors, and performance of social roles and activities in relation to family, work, or independent living [33, 34]. Social inequalities research can also help identify vulnerable groups, including ethnic minority, immigrant, low-income, and socially disadvantaged groups, who are at high risk of disability and uninsurance and who could benefit from public policy and social interventions designed to reduce the impact of disability and uninsurance. Moreover, emphasis on ethnicity and socioeconomic factors is consistent with the national health initiative, *Healthy People 2020*, which calls for further reductions or elimination of social inequalities in health, disease, disability, and access to health services [35]. 

In this study, we use a recent three-year pooled ACS sample containing more than 9 million people to estimate child and adult disability and health insurance rates for detailed racial/ethnic, nativity, and socioeconomic groups in the USA and examine ethnic and nativity patterns after controlling for socioeconomic and demographic characteristics. Additionally, we examine ethnic and socioeconomic disparities in health insurance coverage among people with disabilities. 

## 2. Methods

Data for the present analysis came from the 2008–2010 ACS Micro-data Sample [36]. Decennial censuses conducted by the US Census Bureau have long been the source of detailed socioeconomic and demographic information for various ethnic and immigrant populations in the United States [1]. With the discontinuation of the long-form questionnaire in the 2010 decennial census, the ACS has become the primary census database for producing socioeconomic, demographic, housing, and labor force characteristics of various population groups, including ethnic and immigrant populations, at the national, state, county, and local levels [2, 37]. The advantage of the ACS is that it is conducted annually with a sample size of over 3 million records, as compared with the decennial census long-form data, which were only available every 10 years [37]. The ACS uses a complex, multistage probability design and is representative of the civilian noninstitutionalized population, covering all communities in the USA [36–38]. The household response rate for the 2008–2010 ACS was 98% [2, 38]. All data are based on self-reports and obtained via mail-back questionnaire, telephone, and in-home personal interviews [36, 37]. Substantive and methodological details of the ACS are described elsewhere [36–38]. 

### 2.1. Dependent Variables

Analyses of the two dependent variables, disability and health insurance, were carried out for 9,093,077 individuals, including 2.1 million children aged <18 years. Disability status was a dichotomous variable which defined an individual having a disability if s/he reported serious vision, hearing, cognitive, ambulatory, self-care, or independent living difficulties [36]. The ACS concept of disability captures these six aspects of disability to define an overall measure or specific disability types [18, 19, 36]. To derive *vision-related disability*, the ACS respondents are asked if they are “blind or … have serious difficulty seeing even when wearing glasses.” *Hearing difficulty* is derived from a question that asks respondents if they are “deaf or … have serious difficulty hearing.” *Cognitive difficulty* involves serious difficulty concentrating, remembering, or making decisions due to a physical, metal, or emotion condition. *Ambulatory difficulty* is based on a question that asks respondents if they have “serious difficulty walking or climbing stairs.” *Self-care difficulty* is based on the question whether or not the respondent has difficulty dressing or bathing. *Independent living difficulty* is determined if the respondent reports having difficulty doing errands alone such as visiting a doctor's office or shopping due to a physical, mental, or emotional condition [18, 19, 36]. 

For children under 5 years old, hearing and vision difficulties were used to determine disability status. For children aged 5–14, disability status was determined from hearing, vision, cognitive, ambulatory, and self-care difficulties. For people aged ≥15 years, an individual was considered to have a disability if s/he had difficulty with any one of the 6 disability types [36]. The other dependent variable, health insurance coverage, was also dichotomous. A respondent was considered to have health insurance if s/he reported having any type of private health insurance or public insurance such as Medicaid, Medicare, TRICARE, VA, or Indian Health Service insurance plan [36]. 

### 2.2. Independent Variables

Race/ethnicity was classified into 26 categories as shown in Tables 1–3 and included all of the major racial/ethnic groups such as non-Hispanic Whites, Blacks, American Indians/Alaska Natives, Mexicans, Central/South Americans, Puerto Ricans, Cubans, Asian Indians, Chinese, Filipinos, Japanese, Koreans, Vietnamese, Cambodians, Hawaiians, and some of the newest Asian groups such as Bangladeshis, Pakistanis, Laotians, Thais, and Hmong. With the exception of a residual category of other races that included multiple race groups, all racial/ethnic groups in this study were based on “single race”, indicating that people in these groups indicated only one racial identity [36]. Nativity/immigrant status was defined on the basis of individuals' place of birth [6–8, 36]. US-born people were those born in one of the 50 states, Washington, DC, or US territories. Immigrants or foreign-born people refer to those born outside these areas and who were not a US citizen at birth [6–8, 36]. The joint variable of ethnic immigrant status included 48 categories, with most of the racial/ethnic groups divided into the US-born and foreign-born categories (Tables 4 and 5). Note that American Indians/Alaska Natives, Hawaiians, Samoans, and Guamanians are considered native-born, although a small percentage of people in these groups may have been born outside the USA [36]. 

Using the social determinants of health framework and past research as a guide, we considered, in addition to race/ethnicity and nativity/immigrant status, the following socioeconomic and demographic covariates that are known to be associated with disability and health insurance: age, gender, marital status, and three measures of socioeconomic status (SES): educational attainment, poverty status measured as a ratio of family income to the poverty threshold, and employment status [11–17, 31, 32, 39]. These covariates were measured as shown in Tables 1–3. 

### 2.3. Statistical Methods

Multivariate logistic regression was used to model the association between ethnicity and socioeconomic factors and the binary outcomes of disability and health insurance [40, 41]. The two-sample *t* test was used to test the difference in prevalence between any two groups. Additionally, we used root-mean-square-deviation (RMSD) as a summary measure of ethnic disparities in disability and health insurance coverage [42]. The RMSD is similar to the square root of the variance, except that the average squared deviations are calculated using a “standard” estimate other than the sample mean. The RMSD is given by the formula

(1) $$\begin{array}{c} \text{RMSD}=\text{SQRT}\left\{\sum_{i}\frac{{\left(X_{ri}-X_{rl}\right)}^{2}}{I}\right\}, \end{array}$$

where *X*_*ri*_ is the disability or uninsurance rate for the *i*th group (*i* = 1,2,…, 26), *X*_*rl*_ is the corresponding statistic for the “standard” group (total US population) or group with the lowest rate of disability or uninsurance (i.e., Japanese children or Asian Indian adults), and *I* is the number of ethnic groups (26) being compared.

While RMSD is a measure of absolute health disparity, the coefficient of variation (CV) of the RMSD provides an estimate of relative disparity and is given by

(2) $$\begin{array}{c} \text{CV}\left(\text{RMSD}\right)=\left(\frac{\text{RMSD}}{X_{rl}}\right)\times 100;\;X_{rl}>0. \end{array}$$

## 3. Results

### 3.1. Socioeconomic and Demographic Profiles of Racial/Ethnic Groups

Racial/ethnic groups in the USA vary substantially in their socioeconomic characteristics (Table 1). While Non-Hispanic Whites and the major Asian-American groups such as Asian Indians, Chinese, Filipinos, Japanese, and Koreans had higher education and income levels and lower poverty and unemployment rates, Blacks, American Indians/Alaska Natives, Native Hawaiians, Samoans, Mexican, Puerto Ricans, Central and South Americans, Cambodians, Hmong, and Laotians had substantially lower SES levels. Approximately one third of Black, American Indian/Alaska Native, Hmong, Mexican, and Puerto Rican children were below the poverty line, compared with 5.1% of Filipinos and 6.4% of Japanese children. Approximately 24% of Hmong and American Indian/Alaska Native adults were below the poverty line, compared with 5.6% of Filipino adults. Only 9.3% of Mexicans were college graduates, compared with 71.3% of Asian Indians. More than two thirds of the Asian Indian, Chinese, Filipino, Korean, Vietnamese, Bangladeshi, Pakistani, and Thai populations in the USA were foreign-born, compared with 3.8% of Whites and 7.9% of Blacks. 

### 3.2. Social Inequalities in Disability

**Figure 1:**
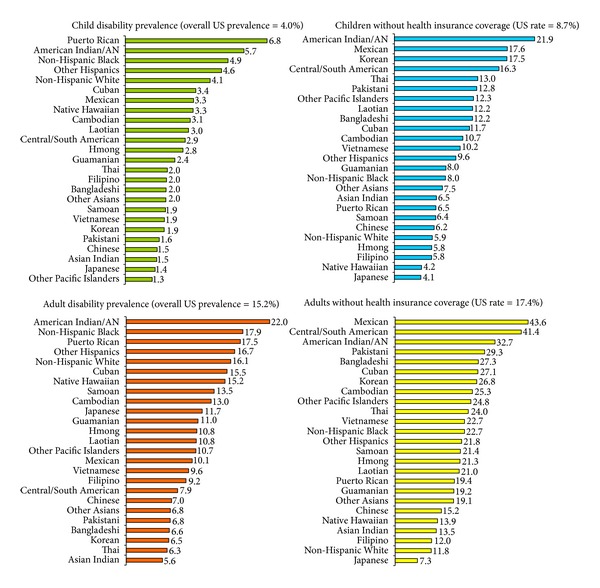
Racial/ethnic disparities in the prevalence (%) of disability and lack of health insurance coverage among US children aged <18 and adults aged 18+ years, 2008–2010.

**Figure 2:**
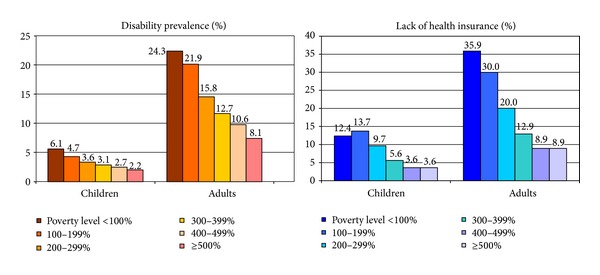
Disparities in prevalence (%) of disability and lack of health insurance coverage among US children aged <18 years and adults aged 18+ years according to poverty level. Notes: Differences in prevalence of disability and health insurance across poverty categories were statistically significant at *P* < 0.001. Source: [36].

During 2008–2010, 12.5%, or 38.4 million people in the US, had a disability. While 4% or 3.0 million children under 18 years of age had a disability, 15.2% or 35.4 million adults had a disability. Disability rates varied from a low of 1.4% for Japanese children and 1.5% for Asian Indian and Chinese children to a high of 5.7% for American Indian/Alaska Native children and 6.8% for Puerto Rican children (Table 2 and Figure 1). The prevalence of disability in adults ranged from 5.6% among Asian Indians to 17.9% among Blacks and 22.0% among American Indians/Alaska Natives (Table 3 and Figure 1). After adjusting for socioeconomic differences, children in almost all Asian and Hispanic subgroups had a significantly lower risk of disability and Puerto Rican children had 42% higher odds of disability than White children (Table 2). While Chinese, Koreans, Japanese, Vietnamese, Asian Indian, Thai, Mexican, and Central/South American adults had lower adjusted odds of disability than Whites, American Indian/Alaska Native adults had 32% higher adjusted odds and Filipino, Cambodian, and Cuban adults 11-12% higher odds than Whites (Table 3). Socioeconomic gradients in disability were marked among both children and adults, with those below the poverty line having 2.2–3.6 times higher odds of disability than their affluent counterparts (Tables 2 and 3 and Figure 2). Adults with less than a high school education had 2.7 times higher adjusted odds of disability than college graduates. The unemployed and those outside the labor force had, respectively, 1.6 and 4.1 times higher adjusted odds than those with a job (Table 3). Differences in socioeconomic characteristics accounted for 60.4% of racial/ethnic variations in child disability and 89.6% of ethnic inequality in adult disability.

### 3.3. Social Inequalities in Health Insurance Coverage

During 2008–2010, 15.3%, or 47.0 million people in the USA, were without health insurance coverage. Approximately 8.7% or 6.4 million children aged <18 lacked health insurance, compared with 17.4% or 40.5 million adults aged ≥18 years. Ethnic disparities in health insurance coverage were at least as pronounced as those in disability. More than 17% of Korean, Mexican, and American Indian/Alaska Native children lacked health insurance, compared with 4.1% of Japanese children and 5.9% of White children (Table 2). Among adults, Mexicans (43.6%), Central/South Americans (41.4%), American Indians/Alaska Natives (32.7%), Pakistanis (29.3%), and Bangladeshis (27.3%) had the highest health uninsurance rates (Table 3 and Figure 1). After adjusting for socioeconomic differences, American Indian/Alaska Native, Mexican, Korean, Central/South American, and Laotian children had 3.5, 2.1, 1.9, 1.8, and 1.4 times higher odds of lacking health insurance coverage than White children, respectively (Table 2). After adjusting for socioeconomic characteristics, American Indian/Alaska Native, Mexican, Korean, Central/South American, and Pakistani adults had 2.2, 1.9, 1.9, 1.7, and 1.5 times higher odds of lacking health insurance coverage than White adults, respectively (Table 3).

Socioeconomic gradients in health insurance coverage among children as well as adults were quite steep, with those below the poverty line having 5-6 times higher adjusted odds of uninsurance than their affluent counterparts. Independent of income levels, adults with less than high school education or without a job had almost 3 times higher odds of lacking health insurance coverage than those with a college degree or a job (Table 3). Socioeconomic differences accounted for 24.4% and 75.1% of racial/ethnic disparities in health insurance coverage among children and adults, respectively.

### 3.4. Ethnic-Nativity Disparities in Disability and Health Insurance

**Figure 3:**
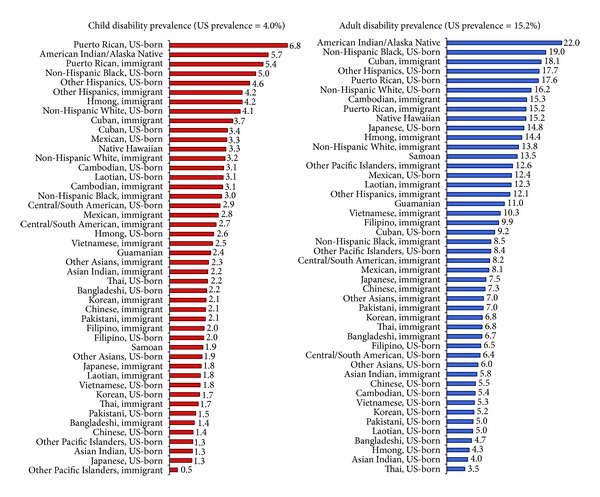
Ethnic immigrant disparities in the prevalence (%) of disability among US children aged <18 years and adults aged 18+ years, 2008–2010.

Ethnic nativity disparities in disability and health insurance coverage were greater than those by race/ethnicity alone (Tables 4 and 5). Although, overall, immigrants had considerably lower disability rates and higher uninsurance rates (Tables 2 and 3), ethnic nativity patterns show the extent of inequalities by immigrant status. While Black, White, and Mexican immigrant children and adults had lower disability rates than their US-born counterparts, immigrant children and adults in most of the Asian subgroups generally had higher disability rates than their US-born counterparts (Tables 4 and 5 and Figure 3). However, children in most ethnic nativity groups, including White and Black immigrant children, had significantly lower risk of disability than US-born White children, even after adjusting for income levels (Table 4). After adjusting for socioeconomic factors, White and Black adult immigrants had 32–42% lower odds of disability and US-born Puerto Ricans and American Indians/Alaska Natives had 7% and 32% higher odds of disability than US-born Whites, respectively (Table 5).

**Figure 4:**
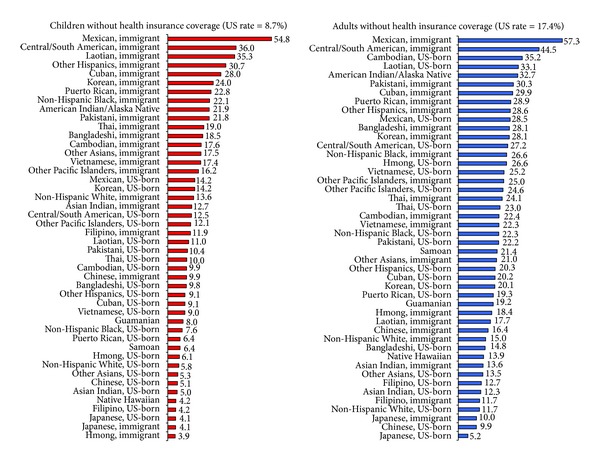
Ethnic immigrant variations in lack of health insurance coverage (%) among US children aged <18 years and adults aged 18+ years, 2008–2010.

Approximately 55% of Mexican immigrant children, 36.0% of Central/South American immigrant children, and 35.2% of Laotian immigrant children lacked health insurance, compared with 4.1% of US- or foreign-born Japanese children and 5.8% of US-born White children (Table 4 and Figure 4). Even after adjusting for socioeconomic differences, Mexican, Central/South American, and Korean immigrant children had 6–11 times higher odds of lacking health insurance coverage than US-born White children (Table 4). Among adults, Mexican immigrants (57.3%), Central/South American immigrants (44.5%), Pakistani immigrants (30.3%), US-born Cambodians (35.2%), US-born Laotians (33.1%), and American Indians/Alaska Natives (32.7%) had the highest uninsurance rates (Table 5 and Figure 4). Socioeconomic characteristics reduced ethnic nativity differences in adult health insurance; however, Mexican, Korean, Central/South American, Cuban, and Pakistani immigrants maintained 3.3–4.5 times higher odds of uninsurance than US-born White adults, respectively (Table 5).

### 3.5. Social Inequalities in Health Insurance among People with Disabilities

Although, overall, people with disabilities were less likely to be uninsured than those without a disability (10.4% versus 16.0%), there were marked ethnic disparities in health insurance coverage among the disabled. More than 20% of Pakistanis, Bangladeshis, American Indians/Alaska Natives, Mexicans, and Central/South Americans with a disability lacked health insurance, compared with 2.3% of Japanese and 8.2% of Whites with disabilities (Table 6). When stratified by nativity status, marked ethnic variations were found in both native- and foreign-born individuals with disabilities (data not shown). For example, >15% of US-born Mexicans and Central/South Americans with disabilities and 34.0% of Mexican immigrants with disabilities were uninsured, compared with 2.0% of US-born Japanese and 6.4% of White immigrants with disabilities. Age, immigrant status, and socioeconomic characteristics largely accounted for racial/ethnic differences in uninsurance among people with disabilities. However, even after adjusting for socioeconomic and demographic differences, American Indians/Alaska Natives, Mexicans, Pakistanis, Central/South Americans, and Asian Indians with disabilities had 1.8, 1.7, 1.6, 1.4, and 1.2 times higher odds of uninsurance than their White counterparts, respectively (Table 6). Strong socioeconomic gradients existed, with people with disabilities in the lowest socioeconomic stratum having 2–4 times higher odds of uninsurance than their affluent counterparts (data not shown).

## 4. Discussion

In this study, we used a large, nationally representative database to examine ethnic and socioeconomic disparities in disability and health insurance in the United States. Because it has a large sample size and is conducted annually, the ACS is an important database for studying and monitoring social inequalities in disability and health insurance coverage in the USA. The new, detailed disability and health insurance statistics for various sociodemographic groups, including those for the newest ethnic immigrant groups, presented herein should serve as the benchmark for setting up national health objectives for various ethnic and immigrant groups in the USA and for conducting further research on the impacts of and factors underlying the disability and health insurance processes. 

Our study reveals considerable ethnic, nativity, and socioeconomic disparities in both disability and health insurance. Among children, Puerto Ricans were at the greatest risk of disability. Although children in many of the Asian subgroups, after the socioeconomic adjustment, had fairly similar risks of disability, they were much less likely to experience disability than their White, Black, American Indian/Alaska Native, and Hispanic counterparts. There was greater heterogeneity in adult disability risks among the Asian subgroups, with Filipinos, Cambodians, Laotians, Hmong, Vietnamese, Asian Indians, and Pakistanis experiencing higher disability risks than Chinese and Koreans. Greater social and economic disadvantage of American Indians/Alaska Natives, Puerto Ricans, and Blacks puts them at a high risk of disabilities, but, even after controlling for SES, they remain at a higher disability risk compared to most other groups. These results are consistent with previous studies [11–17]. 

Among Asians, the Southeast Asian subgroups such as Laotians, Cambodians, Hmong, and Vietnamese are at a higher risk of both child and adult disability, which may partly reflect their immigration circumstances and socioeconomic backgrounds. In contrast to the more affluent Asian groups such as Asian Indians, Chinese, Koreans, and Japanese who have immigrated to the US primarily under skill criteria, many of the Southeast Asian immigrants, substantially less educated and much poorer than other Asian Americans, arrived in the USA under the broad refugee and resettlement act of 1980 [4, 5]. As shown in Table 1, they continue to remain greatly disadvantaged socioeconomically, with Hmong having the highest child and adult poverty rates of all groups in the USA. Native Hawaiians, Samoans, and Guamanians, who are also socioeconomically disadvantaged, have higher adult disability rates than many Asian groups, although they do not differ significantly from Whites or Blacks in their disability risks. All Hispanic subgroups have higher child and adult disability rates than most Asian groups, with Puerto Ricans, the most disadvantaged Hispanic group, showing the highest child disability rate in the US. American Indians/Alaska Natives and Blacks show higher rates of child and adult disability rates than Whites and Asian groups. American Indians/Alaska Natives and Blacks have long experienced a disadvantaged position in the American society, as they have lagged behind Whites in their socioeconomic attainment, employment, health status, and access to and use of health-care services [1, 22, 35]. They are more likely to report higher rates of several health-risk behaviors such as smoking, heavy alcohol consumption, substance use, and co-morbid conditions such as obesity, hypertension, diabetes, and cardiovascular diseases, which may contribute to their higher rates of disability [22, 31, 32, 35]. 

Social inequalities in health insurance coverage were marked, with most of the ethnic inequality attributable to nativity and SES differences. Yet children and adults in several minority and ethnic immigrant groups remained at considerably higher risk of uninsurance compared to Whites of equivalent SES background. An uninsurance rate of >55% for Mexican immigrant children and adults and a rate approaching or exceeding 30% for some of the US-born and low-SES groups indicate the magnitude of the uninsurance problem across various demographic groups in the US.

Since cognitive/mental difficulties contribute most to disabilities in children, differences in socioeconomic, familial, and behavioral risk factors are most likely to explain racial/ethnic and nativity disparities in child disability rates [17, 19]. Since many mental and physical health conditions that cause various disabilities require a doctor's or health-care provider's diagnosis, the substantially lower rates of health insurance, healthcare access, and healthcare utilization or interaction with the healthcare system among immigrant and ethnic minority groups such as Asians, Hispanics, Blacks, and American Indians/Alaska Natives might result in fewer diagnoses of disability-related health conditions among them and could partly account for the racial/ethnic disparities in disability rates reported here. Asian and Hispanic subgroups vary greatly in their cultures, English language proficiencies, and perhaps in their interpretation/understanding of the disability questions in the ACS, all of which can also contribute to the reported ethnic differences in disability rates. Among people with disabilities, American Indians/Alaska Natives, Hispanics, Asians, and several immigrant groups have substantially high rates of uninsurance, which may imply that they are more likely to delay or not receive needed medical care, preventive health services, social, and/or rehabilitative services [22, 23]. 

Ethnic and nativity patterns in disability are consistent with those observed for a wide range of health outcomes [11, 12, 22, 29, 31, 32, 42]. In the USA, the major Asian and Hispanic subgroups, including Chinese, Asian Indians, Filipinos, Japanese, Mexicans, Cubans, and Central/South Americans have higher life expectancy and lower rates of all-cause, chronic disease, and injury mortality and morbidity than Whites, Blacks, and American Indians/and Alaska Natives [11, 12, 22, 29, 31, 32]. Consistent with the patterns in disability, immigrants, overall and in most racial/ethnic groups, do better than the US-born in various child and adult health outcomes [11, 12, 29, 42]. Healthy immigrant effect or positive immigrant selectivity (i.e., people immigrating to the USA may be healthier than those who remain in their countries of origin) has been offered as an explanation of better health and lower disability and mortality rates of immigrants [11–13, 29, 42]. Acculturation, often measured by duration of residence since the time of immigration, is also shown to play a part in modifying the risks of disability and health insurance among immigrants [11–13]. However, lower disability rates among individuals in many of the US-born Asian and Hispanic subgroups compared with US-born Whites may offer support for the cultural pluralism hypothesis, which contends that many groups retain significant ethnic and social ties to their cultural heritage across generations in the host country [11, 12, 43]. 

Our study has limitations. Although the ACS summary database does include the type of disability (sensory, mental, and physical) for both children and adults, the micro-data sample lacks data on both the type and severity of disabilities [2, 18, 19, 36]. Social and ethnic patterns might vary according to disability type and may be more pronounced in physical than mental health disabilities [13–19]. The ACS also excludes data on institutionalized populations, such as those in prisons, nursing homes, and military who may have different disability and uninsurance rates than the general population [36–38]. Additionally, although the ACS does include a number of immigration-related variables such as citizenship/naturalization status, English language ability, length of US residence, and age at entry into the US, it does lack data on the legal status of immigrants which could greatly influence their access to health insurance [36–38]. Lastly, the ACS is a cross-sectional database, and disability can be both a cause and consequence of social and economic disadvantage [25, 36, 37]. 

Although the definition of disability varies somewhat across the developed world, the disability rates in the USA are comparable to those in Canada, Australia, and the United Kingdom [21, 25, 44, 45]. However, the social, economic, and labor force experiences of people with disabilities, and disability policies vary greatly among nations. According to a recent Organisation for Economic Cooperation and Development (OECD) study, young Americans aged 18–29 with a disability were 80% less likely to be employed than their counterparts without a disability [45]. The ACS data show an adult employment rate of 21.8% for people with disabilities, compared with 64.2% for people without a disability [2]. Of the 25 OECD countries, the gap in employment by disability status and the poverty rate for households with a disabled child were the highest in the USA [45]. The USA ranks poorly in its social and economic inclusion of people with disabilities and in its disability benefit, compensation, and integration policies compared to most other OECD nations, particularly the Nordic countries [45]. 

Children with disabilities from immigrant families confront challenges in access to high-quality medical care due to lack of parental awareness in eligibility criteria for safety-net programs and insurance and nativity status of the parent [46, 47]. Policymakers have made advances in children's health insurance coverage by passing the Children's Health Insurance Program Reauthorization Act (CHIPRA) of 2009, which granted states an option to provide federally funded Medicaid and CHIP coverage for lawfully residing children and pregnant women. Although twenty-three states and the District of Columbia are using this option to offer coverage to legal immigrant children without a five-year waiting period, children residing in states that have not elected to implement eligibility expansion or to simplify enrollment and renewal procedures remain at risk for uninsurance [48]. Inadequate access to quality care for these children warrants further policy solutions to improve their health care utilization, especially in obtaining culturally sensitive care, community-based support, and advocacy for services [49–51]. 

For adults, the Affordable Care Act of 2010 will offer options for legal immigrants to purchase affordable coverage through the Health Insurance Marketplace (also known as the Exchange). Initial provision of the Affordable Care Act has provided coverage to millions of young adults by permitting them to stay on their parents' health plan until age 26 and children with pre-existing conditions by requiring insurers to no longer exclude, limit, or deny coverage to children under age 19 solely based on a health problem or disability [52]. Moreover, the Health Insurance Marketplace will be a new pathway to purchase health insurance beginning on October 1, 2013. Families will be able to get financial assistance through the Health Insurance Marketplace. There will be new, expanded programs available, and more people than ever before will qualify for free or low-cost health insurance programs. Concurrently, the federally funded Marketplace Navigators program will provide culturally and linguistically appropriate consumer information and assistance regarding public and private insurance coverage to diverse communities and people with disabilities [53]. This program will provide critical family support in navigation through the Health Insurance Marketplace. 

The findings presented here demonstrate considerable heterogeneity in disability and insurance status among racial/ethnic, immigrant, and socioeconomic groups. While the provisions of the Affordable Care Act hold promise for expanding coverage to those currently uninsured, targeted and culturally competent outreach and enrollment programs for the Health Insurance Marketplace will be critical in raising public awareness as racial/ethnic and immigrant groups may have different levels of awareness and/or understanding about benefits and eligibility criteria of health insurance plans and safety net programs. The successful outreach of the Marketplace Navigators and other in-person assistance programs through initiating new or enhancing existing partnerships with ethnic immigrant community-based organizations will greatly benefit ethnic immigrant groups, especially individuals and children with disabilities.

## Figures and Tables

**Table 1:** Descriptive socioeconomic and demographic characteristics for major racial/ethnic groups in the United States: the 2008–2010 American Community Survey (*N* = 9,093,077).

Racial/ethnic group	Number in sample	Population proportion (%)	Child poverty^1^ rate (%)	Adult poverty^2^ rate (%)	Per capita income^2^ ($)	College graduates^3^ (%)	Unemployment rate^2^ (%)	Immigrant population (%)	Divorced or separated^2^ (%)
*United States *	9,093,077	100.00	19.96	12.48	36,498	28.03	8.63	12.82	13.54
Non-Hispanic White	6,335,580	64.14	11.97	9.42	39,964	31.11	7.20	3.84	13.53
Mexican	780,939	10.45	32.10	20.65	22,171	9.25	10.25	36.19	10.99
Puerto Rican	108,418	1.49	32.11	21.60	26,896	16.26	12.99	1.10	16.51
Cuban	46,579	0.57	16.05	15.80	30,992	24.54	10.67	58.87	16.45
Central/South American	196,267	2.79	24.21	16.80	25,543	18.71	9.95	64.02	13.22
Other Hispanics	65,309	0.77	22.95	15.34	30,267	21.29	9.80	13.42	15.51
Non-Hispanic Black	903,942	12.23	36.00	21.40	25,155	17.82	14.54	7.85	17.53
American Indian/AN	68,673	0.66	32.45	23.78	23,721	13.85	14.29	1.10	17.63
Asian Indian	72,611	0.90	7.11	8.74	53,639	71.25	6.72	72.19	3.32
Chinese	95,691	1.09	10.40	13.62	42,567	52.60	6.50	69.24	5.87
Filipino	71,631	0.82	5.09	5.67	36,745	48.64	6.58	66.67	8.20
Japanese	23,414	0.25	6.44	8.27	44,550	47.55	4.02	41.15	9.70
Korean	38,039	0.47	13.50	15.35	35,604	52.02	7.03	73.91	6.65
Vietnamese	42,879	0.51	16.54	14.10	29,415	26.14	8.20	68.79	7.84
Cambodian	5,782	0.08	24.64	17.04	22,500	13.65	11.39	59.51	9.39
Bangladeshi	2,523	0.03	28.68	21.15	26,887	49.83	7.95	73.50	2.50
Pakistani	8,256	0.11	19.92	14.11	38,113	54.43	8.18	67.54	3.59
Hmong	4,947	0.08	35.65	24.23	18,411	12.90	12.21	43.72	5.63
Laotian	4,545	0.06	16.50	13.34	24,735	13.10	11.48	59.28	10.34
Thai	4,384	0.05	24.07	15.94	28,581	43.73	6.73	77.28	10.58
Other Asians	22,412	0.26	14.81	14.74	35,064	45.45	8.04	58.00	6.16
Native Hawaiian	4,277	0.05	15.26	12.31	31,095	16.86	10.44	1.82	14.99
Samoan	2,101	0.03	21.42	15.75	22,731	11.32	14.28	13.82	8.19
Guamanian	1,612	0.02	14.46	10.72	33,138	18.09	9.23	1.52	12.40
Other Pacific Islanders	1,736	0.02	17.53	15.23	25,191	16.03	13.59	38.29	8.33
All other groups^4^	180,530	2.09	19.96	15.56	30,345	28.01	11.88	9.96	14.82

^1^Children under 18 years of age. ^2^Population aged 18 years and older. ^3^Population aged 25 years and older. ^4^This category includes multiple race groups. AN: Alaska Native.

**Table 2:** Prevalence, unadjusted, and adjusted odds of disability and lack of health insurance among US children under 18 years of age according to racial/ethnic, socioeconomic, and demographic characteristics: the 2008–2010 American Community Survey (*N* = 2,079,138).

Racial/ethnic and socioeconomic groups	Disability						No health insurance					
	Prevalence		Unadj.odds ratio		Adjusted odds ratio^1^		Prevalence		Unadj. odds ratio		Adjusted odds ratio^1^	
	%	SE	OR	95% CI	OR	95% CI	%	SE	OR	95% CI	OR	95% CI
*Race/ethnicity *												
Non-Hispanic White	4.08	0.02	1.00	Reference	1.00	Reference	5.89	0.02	1.00	Reference	1.00	Reference
Mexican	3.28	0.03	0.87	0.85–0.89	0.69	0.67–0.71	17.60	0.07	3.39	3.35–3.44	2.12	2.09–2.14
Puerto Rican	6.82	0.13	1.78	1.71–1.86	1.42	1.36–1.48	6.46	0.13	1.11	1.06–1.16	0.87	0.83–0.91
Cuban	3.39	0.19	0.89	0.80–0.99	0.89	0.80–1.00	11.71	0.30	1.93	1.80–2.06	1.35	1.26–1.45
Central/South American	2.88	0.07	0.77	0.74–0.81	0.70	0.66–0.73	16.29	0.15	3.09	3.01–3.16	1.81	1.76–1.86
Other Hispanics	4.63	0.15	1.20	1.12–1.28	1.02	0.96–1.09	9.57	0.20	1.81	1.73–1.90	1.43	1.37–1.51
Non-Hispanic Black	4.94	0.05	1.44	1.41–1.47	1.04	1.02–1.06	7.95	0.06	1.47	1.44–1.49	1.01	1.00–1.03
American Indian/AN	5.71	0.17	1.41	1.33–1.51	1.03	0.97–1.10	21.86	0.30	4.74	4.58–4.92	3.51	3.38–3.64
Asian Indian	1.51	0.09	0.39	0.35–0.44	0.57	0.51–0.64	6.54	0.17	1.04	0.97–1.10	0.94	0.88–1.01
Chinese	1.53	0.09	0.41	0.37–0.46	0.49	0.44–0.55	6.19	0.16	1.02	0.96–1.08	0.77	0.72–0.82
Filipino	1.98	0.12	0.51	0.45–0.57	0.59	0.52–0.66	5.79	0.20	1.06	0.99–1.14	0.72	0.67–0.78
Japanese	1.43	0.26	0.41	0.30–0.56	0.52	0.38–0.71	4.12	0.38	0.68	0.55–0.84	0.54	0.44–0.67
Korean	1.87	0.16	0.48	0.41–0.56	0.52	0.44–0.62	17.54	0.41	3.22	3.02–3.42	1.87	1.75–2.00
Vietnamese	1.88	0.14	0.53	0.46–0.60	0.50	0.44–0.57	10.24	0.28	1.77	1.66–1.89	1.13	1.06–1.22
Cambodian	3.12	0.49	0.97	0.75–1.25	0.75	0.58–0.98	10.66	0.73	1.77	1.49–2.10	1.08	0.90–1.29
Bangladeshi	1.96	0.52	0.53	0.33–0.86	0.49	0.30–0.80	12.18	1.15	2.33	1.87–2.89	1.08	0.86–1.35
Pakistani	1.63	0.27	0.46	0.35–0.62	0.46	0.35–0.61	12.83	0.61	2.17	1.92–2.45	1.25	1.10–1.42
Hmong	2.77	0.35	0.67	0.52–0.88	0.49	0.38–0.64	5.81	0.53	1.19	1.00–1.42	0.57	0.47–0.68
Laotian	3.04	0.52	0.82	0.60–1.13	0.65	0.47–0.90	12.19	0.90	2.11	1.75–2.54	1.43	1.18–1.73
Thai	2.02	0.59	0.59	0.36–0.98	0.57	0.34–0.93	13.00	1.29	2.54	2.02–3.19	1.22	0.95–1.55
Other Asians	1.95	0.17	0.52	0.44–0.61	0.60	0.50–0.70	7.48	0.31	1.29	1.17–1.42	0.90	0.81–0.99
Native Hawaiian	3.26	0.58	0.85	0.60–1.20	0.70	0.49–0.99	4.22	0.64	0.76	0.55–1.04	0.61	0.45–0.84
Samoan	1.93	0.62	0.64	0.39–1.03	0.45	0.28–0.73	6.44	0.93	1.13	0.82–1.56	0.76	0.55–1.05
Guamanian	2.38	0.91	0.76	0.42–1.38	0.64	0.35–1.17	8.03	1.43	1.54	1.05–2.25	1.29	0.88–1.88
Other Pacific Islanders	1.29	0.55	0.43	0.23–0.81	0.36	0.19–0.67	12.33	1.29	2.07	1.59–2.71	1.43	1.09–1.89
All other groups^2^	5.00	0.08	1.28	1.24–1.32	1.25	1.21–1.30	6.42	0.08	1.13	1.10–1.16	1.02	0.99–1.05

*Poverty status (ratio of family income to poverty threshold) *												
<100%	6.09	0.04	3.12	3.05–3.20	3.62	3.53–3.72	12.37	0.05	6.85	6.68–7.02	5.50	5.36–5.64
100–199%	4.67	0.03	2.29	2.23–2.35	2.54	2.47–2.60	13.73	0.05	7.54	7.36–7.72	6.24	6.09–6.40
200–299%	3.64	0.03	1.72	1.67–1.76	1.79	1.75–1.84	9.65	0.05	5.01	4.89–5.14	4.57	4.46–4.69
300–399%	3.11	0.03	1.43	1.39–1.47	1.45	1.41–1.50	5.62	0.04	2.75	2.67–2.82	2.65	2.58–2.72
400–499%	2.71	0.04	1.24	1.20–1.28	1.25	1.20–1.29	3.59	0.04	1.67	1.62–1.73	1.65	1.60–1.71
≥500%	2.20	0.02	1.00	Reference	1.00	Reference	2.14	0.02	1.00	Reference	1.00	reference
Unknown	10.83	0.19	6.34	6.09–6.59	6.56	6.30–6.84	12.39	0.18	6.86	6.58–7.15	6.05	5.80–6.31

*Age (years) *												
0–5	1.24	0.01	1.00	Reference	1.00	Reference	8.45	0.03	1.00	Reference	1.00	Reference
6–11	5.20	0.03	4.48	4.37–4.59	4.69	4.57–4.81	10.47	0.03	1.20	1.18–1.21	1.18	1.17–1.20
12–17	5.60	0.03	4.91	4.79–5.03	5.33	5.20–5.47	7.08	0.03	1.53	1.51–1.55	1.54	1.52–1.56

*Gender *												
Male	5.00	0.02	1.67	1.65–1.70	1.68	1.66–1.70	8.70	0.03	1.00	0.99–1.01	1.00	0.99–1.01
Female	3.05	0.02	1.00	Reference	1.00	Reference	8.70	0.03	1.00	Reference	1.00	Reference

*Immigrant status *												
Immigrant	2.78	0.06	0.74	0.71–0.78	0.66	0.63–0.69	32.16	0.17	5.21	5.12–5.30	3.66	3.59–3.74
US-born	4.10	0.01	1.00	Reference	1.00	Reference	7.76	0.02	1.00	Reference	1.00	Reference

OR: odds ratio; SE: standard error; CI: confidence interval; AN: Alaska Native. ^1^Adjusted by logistic regression model for age, gender, race/ethnicity, immigrant status, and poverty status. ^2^This category includes multiple race groups.

**Table 3:** Prevalence, unadjusted, and adjusted odds of disability and lack of health insurance among US adults aged 18+ years according to racial/ethnic, socioeconomic, and demographic characteristics: the 2008–2010 American Community Survey (*N* = 7,013,939).

Racial/ethnic and socioeconomic groups	Disability						No health insurance					
	Prevalence		Unadjusted odds ratio		Adjusted odds ratio^1^		Prevalence		Unadjusted odds ratio		Adjusted odds ratio^1^	
	%	SE	OR	95% CI	OR	95% CI	%	SE	OR	95% CI	OR	95% CI
*Race/ethnicity *												
Non-Hispanic White	16.05	0.02	1.00	Reference	1.00	Reference	11.82	0.01	1.00	Reference	1.00	Reference
Mexican	10.13	0.05	0.65	0.65–0.66	0.83	0.82–0.84	43.61	0.07	5.66	5.63–5.70	1.87	1.85–1.89
Puerto Rican	17.52	0.14	1.15	1.13–1.17	1.06	1.04–1.09	19.41	0.14	1.92	1.89–1.96	0.92	0.91–0.94
Cuban	15.48	0.19	1.00	0.97–1.03	1.12	1.08–1.15	27.10	0.22	2.59	2.53–2.66	1.46	1.42–1.51
Central/South American	7.89	0.08	0.49	0.48–0.49	0.85	0.83–0.87	41.35	0.13	5.11	5.05–5.17	1.65	1.63–1.68
Other Hispanics	16.65	0.18	1.11	1.09–1.14	1.17	1.13–1.20	21.80	0.19	2.14	2.09–2.19	1.25	1.22–1.28
Non-Hispanic Black	17.91	0.05	1.28	1.27–1.29	1.02	1.02–1.03	22.72	0.05	2.34	2.32–2.35	1.11	1.10–1.12
American Indian/AN	22.03	0.19	1.56	1.53–1.59	1.32	1.29–1.36	32.73	0.21	3.92	3.84–4.00	2.20	2.15–2.24
Asian Indian	5.57	0.10	0.31	0.30–0.32	0.94	0.90–0.98	13.46	0.14	1.20	1.17–1.24	0.86	0.84–0.89
Chinese	6.97	0.09	0.39	0.38–0.41	0.66	0.64–0.68	15.15	0.12	1.37	1.34–1.40	0.80	0.78–0.82
Filipino	9.17	0.12	0.52	0.51–0.53	1.16	1.13–1.20	11.95	0.13	1.07	1.04–1.10	0.77	0.75–0.79
Japanese	11.66	0.23	0.75	0.72–0.78	0.80	0.77–0.84	7.29	0.16	0.56	0.53–0.60	0.63	0.59–0.67
Korean	6.54	0.15	0.38	0.37–0.40	0.67	0.64–0.71	26.75	0.25	2.83	2.76–2.91	1.85	1.80–1.91
Vietnamese	9.58	0.17	0.57	0.55–0.59	0.92	0.88–0.95	22.73	0.23	2.30	2.24–2.37	0.90	0.87–0.93
Cambodian	13.03	0.52	0.76	0.70–0.83	1.11	1.01–1.23	25.33	0.65	2.70	2.51–2.89	0.76	0.70–0.82
Bangladeshi	6.61	0.60	0.35	0.29–0.42	0.86	0.70–1.05	27.26	1.04	2.98	2.67–3.32	1.11	0.99–1.25
Pakistani	6.75	0.33	0.36	0.33–0.40	0.91	0.82–1.02	29.33	0.58	3.13	2.95–3.32	1.54	1.44–1.65
Hmong	10.84	0.59	0.64	0.57–0.71	1.06	0.93–1.20	21.26	0.77	2.39	2.18–2.61	0.56	0.51–0.62
Laotian	10.75	0.56	0.68	0.61–0.75	1.07	0.96–1.20	20.98	0.68	2.15	1.98–2.35	0.65	0.59–0.71
Thai	6.28	0.41	0.36	0.31–0.41	0.70	0.61–0.80	23.96	0.67	2.41	2.23–2.61	1.21	1.11–1.32
Other Asians	6.78	0.21	0.38	0.36–0.41	0.82	0.76–0.87	19.14	0.30	1.81	1.74–1.89	0.82	0.78–0.86
Native Hawaiian	15.17	0.65	1.01	0.92–1.11	1.06	0.96–1.18	13.90	0.59	1.38	1.25–1.53	0.88	0.79–0.98
Samoan	13.49	0.91	0.81	0.70–0.94	1.16	0.98–1.36	21.38	1.05	2.20	1.93–2.50	0.86	0.75–0.99
Guamanian	11.02	0.92	0.67	0.57–0.80	0.99	0.82–1.20	19.23	1.04	1.70	1.46–1.97	1.07	0.90–1.26
Other Pacific Islanders	10.70	0.91	0.60	0.50–0.73	0.93	0.76–1.14	24.83	1.23	2.60	2.27–2.98	0.98	0.84–1.13
All other groups^2^	17.38	0.13	1.18	1.16–1.20	1.65	1.62–1.68	21.04	0.12	2.09	2.05–2.12	1.17	1.15–1.19

*Education (years of school completed) *												
0–11	28.62	0.05	5.53	5.49–5.57	2.74	2.72–2.77	32.28	27.07	6.26	6.21–6.31	2.99	2.96–3.02
12	18.31	0.03	2.90	2.88–2.91	1.74	1.73–1.76	21.13	17.58	3.60	3.57–3.63	2.41	2.39–2.43
13–15	12.23	0.02	1.78	1.77–1.80	1.53	1.52–1.54	15.59	13.33	2.59	2.57–2.61	1.66	1.64–1.67
≥16	7.46	0.02	1.00	Reference	1.00	Reference	6.76	5.60	1.00	Reference	1.00	Reference

*Poverty status (ratio of family income to poverty threshold) *												
<100%	24.30	0.05	4.08	4.05–4.11	2.27	2.25–2.29	35.85	0.05	12.13	12.03–12.24	5.62	5.56–5.67
100–199%	21.86	0.04	3.57	3.54–3.59	1.91	1.90–1.93	29.97	0.04	8.62	8.55–8.69	5.46	5.41–5.51
200–299%	15.78	0.04	2.32	2.30–2.33	1.50	1.49–1.51	20.04	0.04	5.04	5.00–5.08	3.41	3.38–3.44
300–399%	12.69	0.03	1.75	1.74–1.76	1.33	1.32–1.34	12.94	0.03	2.98	2.95–3.01	2.18	2.16–2.20
400–499%	10.62	0.04	1.40	1.39–1.41	1.21	1.20–1.22	8.92	0.03	1.96	1.94–1.98	1.55	1.54–1.57
≥500%	8.05	0.02	1.00	Reference	1.00	Reference	4.76	0.01	1.00	Reference	1.00	Reference
Unknown	32.93	0.11	7.17	7.09–7.24	3.27	3.23–3.31	26.84	0.10	10.53	10.41–10.65	5.91	5.83–6.00

*Employment status *												
Unemployed	11.00	0.05	2.10	2.08–2.12	1.68	1.66–1.70	46.18	0.08	4.72	4.68–4.75	2.88	2.86–2.91
Not in labor force	33.41	0.03	8.45	8.41–8.49	4.10	4.08–4.12	14.17	0.02	0.84	0.84-0.84	1.00	1.00–1.01
Employed	5.59	0.01	1.00	Reference	1.00	Reference	16.48	0.02	1.00	Reference	1.00	Reference

*Age (years) *												
18–24	5.58	0.03	1.00	Reference	1.00	Reference	29.31	0.05	3.24	3.22–3.27	1.43	1.42–1.45
25–34	5.81	0.02	1.00	0.99–1.01	1.98	1.95–2.00	27.48	0.04	2.79	2.77–2.81	2.01	1.99–2.02
35–44	7.91	0.02	1.35	1.33–1.37	3.28	3.24–3.33	20.48	0.04	1.87	1.85–1.88	1.52	1.50–1.53
45–54	12.81	0.03	2.28	2.26–2.31	5.83	5.75–5.90	16.35	0.03	1.45	1.44–1.46	1.34	1.33–1.36
55–64	19.06	0.04	3.67	3.63–3.71	7.75	7.65–7.85	11.80	0.03	1.00	Reference	1.00	Reference
≥65	39.20	0.04	10.13	10.03–10.24	9.64	9.52–9.77	0.95	0.01	0.06	0.06-0.06	0.04	0.04-0.04

*Gender *												
Male	14.56	0.02	0.93	0.92–0.93	1.27	1.27–1.28	20.03	0.02	1.41	1.40–1.42	1.37	1.36–1.38
Female	15.82	0.02	1.00	Reference	1.00	Reference	14.96	0.02	1.00	Reference	1.00	Reference

*Immigrant status *												
Immigrant	9.55	0.03	0.58	0.57–0.58	0.60	0.60–0.61	33.61	0.05	2.85	2.84–2.86	2.15	2.13–2.16
US-born	16.26	0.02	1.00	Reference	1.00	Reference	14.40	0.01	1.00	Reference	1.00	Reference

*Marital status *												
Married	12.05	0.02	1.00	Reference	1.00	Reference	11.40	0.01	1.00	Reference	1.00	Reference
Widowed	47.25	0.07	6.08	6.04–6.12	1.91	1.90–1.93	5.71	0.03	0.46	0.45–0.47	1.56	1.53–1.58
Divorced/separated	20.97	0.04	1.88	1.87–1.89	1.56	1.55–1.57	22.29	0.04	2.46	2.44–2.47	1.93	1.91–1.94
Never married	10.94	0.03	0.91	0.90–0.91	1.66	1.65–1.68	29.09	0.03	3.60	3.58–3.62	1.88	1.86–1.89

OR: odds ratio; SE: standard error; CI: confidence interval; AN: Alaska Native.

^1^Adjusted by logistic regression model for age, gender, race/ethnicity, immigrant status, marital status, education, poverty, and employment status.

^2^This category includes multiple race groups.

**Table 4:** Prevalence and adjusted odds of disability and lack of health insurance among US children under 18 years of age in 48 ethnic immigrant groups: the 2008–2010 American Community Survey (*N* = 2,079,138).

	Disability					No health insurance				
Ethnic immigrant group	Prevalence		Adjusted odds ratio^1^			Prevalence		Adjusted odds ratio^1^		
	%	SE	OR	95% CI		%	SE	OR	95% CI	
Non-Hispanic White, US-born	4.09	0.02	1.00	Reference		5.81	0.02	1.00	Reference	
Non-Hispanic White, immigrant	3.21	0.17	0.50	0.37	0.68	13.64	0.28	1.97	1.66	2.33
Mexican, US-born	3.32	0.04	0.70	0.64	0.76	14.23	0.07	2.13	2.01	2.25
Mexican, immigrant	2.82	0.12	0.42	0.34	0.52	54.76	0.33	10.70	9.81	11.68
Puerto Rican, US-born	6.82	0.13	1.25	1.07	1.47	6.44	0.13	1.02	0.87	1.20
Puerto Rican, immigrant	5.41	3.32	5.07	0.91	28.20	22.82	4.48	4.24	0.78	23.21
Cuban, US-born	3.35	0.20	0.60	0.36	0.99	9.05	0.29	1.71	1.25	2.34
Cuban, immigrant	3.66	0.54	0.62	0.27	1.42	28.04	1.26	3.20	2.13	4.81
Central/South American, US-born	2.92	0.08	0.63	0.51	0.78	12.49	0.15	2.05	1.81	2.32
Central/South American, immigrant	2.69	0.18	0.37	0.25	0.53	36.04	0.49	5.61	4.89	6.43
Other Hispanics, US-born	4.64	0.15	1.15	0.93	1.44	9.05	0.20	1.27	1.04	1.55
Other Hispanics, immigrant	4.20	0.92	0.99	0.35	2.78	30.65	1.94	3.14	1.63	6.06
Non-Hispanic Black, US-born	4.99	0.05	0.98	0.91	1.05	7.60	0.05	1.16	1.09	1.23
Non-Hispanic Black, immigrant	3.01	0.24	0.64	0.44	0.93	22.08	0.53	3.22	2.65	3.91
American Indian/Alaska Native	5.71	0.17	1.07	0.87	1.32	21.86	0.30	3.56	3.11	4.07
Asian Indian, US-born	1.32	0.09	0.33	0.16	0.66	4.95	0.17	1.31	0.91	1.88
Asian Indian, immigrant	2.21	0.26	0.34	0.15	0.77	12.65	0.52	3.24	2.38	4.40
Chinese, US-born	1.36	0.10	0.46	0.29	0.72	5.07	0.17	0.96	0.71	1.31
Chinese, immigrant	2.08	0.23	0.20	0.08	0.49	9.88	0.40	2.56	1.91	3.44
Filipino, US-born	1.97	0.14	0.60	0.37	0.96	4.15	0.19	1.04	0.73	1.48
Filipino, immigrant	2.00	0.26	0.62	0.34	1.14	11.93	0.57	2.43	1.76	3.36
Japanese, US-born	1.29	0.28	0.52	0.16	1.65	4.12	0.42	1.57	0.75	3.26
Japanese, immigrant	1.83	0.58	#			4.10	0.84	0.93	0.22	3.96
Korean, US-born	1.74	0.19	0.63	0.34	1.16	14.21	0.47	3.65	2.70	4.93
Korean, immigrant	2.11	0.29	0.24	0.09	0.63	23.99	0.80	5.84	4.40	7.75
Vietnamese, US-born	1.77	0.15	0.49	0.29	0.82	9.03	0.28	1.76	1.34	2.33
Vietnamese, immigrant	2.49	0.43	0.33	0.12	0.90	17.37	0.91	2.57	1.73	3.81
Cambodian, US-born	3.13	0.52	0.44	0.16	1.21	9.90	0.75	1.06	0.57	1.98
Cambodian, immigrant	3.07	1.42	1.60	0.20	12.82	17.61	2.63	2.99	0.62	14.51
Bangladeshi, US-born	2.15	0.63	#			9.81	1.21	9.96	2.94	33.74
Bangladeshi, immigrant	1.43	0.92	#			18.46	2.66	6.24	2.21	17.61
Pakistani, US-born	1.51	0.30	0.63	0.20	2.02	10.39	0.64	1.70	0.83	3.48
Pakistani, immigrant	2.07	0.58	0.21	0.03	1.53	21.84	1.62	4.40	2.51	7.71
Hmong, US-born	2.55	0.36	0.31	0.10	0.99	6.12	0.60	0.61	0.28	1.31
Hmong, immigrant	4.20	1.24	1.44	0.43	4.85	3.87	1.02	0.35	0.05	2.58
Laotian, US-born	3.10	0.53	1.03	0.41	2.56	11.03	0.89	1.73	0.88	3.39
Laotian, immigrant	1.78	2.34	#			35.32	5.75	4.90	0.86	28.03
Thai, US-born	2.20	0.73	0.64	0.09	4.73	10.01	1.34	2.43	0.82	7.17
Thai, immigrant	1.66	1.01	#			19.02	2.74	3.17	1.34	7.47
Other Asians, US-born	1.88	0.19	0.48	0.23	1.03	5.27	0.29	1.16	0.72	1.89
Other Asians, immigrant	2.25	0.48	0.23	0.06	0.95	17.48	1.07	1.81	1.03	3.15
Native Hawaiian	3.26	0.58	0.20	0.03	1.45	4.22	0.64	0.89	0.36	2.23
Samoan	1.93	0.62	0.72	0.22	2.35	6.44	0.93	1.05	0.42	2.67
Guamanian	2.38	0.91	0.83	0.11	6.33	8.03	1.44	2.46	0.69	8.75
Other Pacific Islanders, US-born	1.34	0.56	1.13	0.27	4.83	12.07	1.31	1.89	0.64	5.53
Other Pacific Islanders, immigrant	0.45	2.44	#			16.17	5.95	#		
All other groups, US-born	5.02	0.08	1.49	1.33	1.67	6.20	0.08	1.03	0.91	1.17
All other groups, immigrant	4.21	0.52	0.19	0.05	0.76	17.76	0.96	2.39	1.54	3.72

OR: odds ratio; SE: standard error; CI: confidence interval. #: Insufficient data.

^1^Adjusted by logistic regression model for age, gender, and poverty/family income levels.

**Table 5:** Prevalence and adjusted odds of disability and lack of health insurance among US adults aged 18+ years in 48 ethnic immigrant groups: the 2008–2010 American Community Survey (*N* = 7,013,939).

Ethnic immigrant group	Disability					No health insurance				
	Prevalence		Adjusted odds ratio^1^			Prevalence		Adjusted odds ratio^1^		
	%	SE	OR	95% CI		%	SE	OR	95% CI	
Non-Hispanic White, US-born	16.16	0.02	1.00	Reference		11.67	0.01	1.00	Reference	
Non-Hispanic White, immigrant	13.75	0.08	0.68	0.67	0.69	15.02	0.07	1.78	1.75	1.81
Mexican, US-born	12.40	0.07	0.92	0.91	0.94	28.53	0.09	1.61	1.59	1.62
Mexican, immigrant	8.07	0.06	0.44	0.43	0.45	57.26	0.10	4.51	4.47	4.56
Puerto Rican, US-born	17.56	0.15	1.07	1.04	1.09	19.25	0.14	0.92	0.90	0.94
Puerto Rican, immigrant	15.18	1.09	0.61	0.51	0.73	28.87	1.29	1.82	1.57	2.10
Cuban, US-born	9.19	0.28	0.99	0.92	1.06	20.24	0.37	1.27	1.20	1.34
Cuban, immigrant	18.08	0.25	0.70	0.67	0.72	29.93	0.27	3.29	3.18	3.40
Central/South American, US-born	6.38	0.16	0.80	0.76	0.84	27.22	0.27	1.46	1.41	1.50
Central/South American, immigrant	8.22	0.09	0.52	0.51	0.53	44.47	0.14	3.61	3.56	3.66
Other Hispanics, US-born	17.69	0.21	1.19	1.16	1.23	20.25	0.20	1.28	1.24	1.32
Other Hispanics, immigrant	12.10	0.38	0.64	0.59	0.68	28.60	0.48	2.38	2.25	2.53
Non-Hispanic Black, US-born	18.96	0.05	1.03	1.02	1.04	22.29	0.05	1.13	1.12	1.14
Non-Hispanic Black, immigrant	8.46	0.12	0.58	0.57	0.60	26.62	0.17	1.99	1.95	2.03
American Indian/AN	22.03	0.19	1.32	1.29	1.35	32.73	0.21	2.20	2.15	2.25
Asian Indian, US-born	4.00	0.26	0.63	0.55	0.72	12.33	0.42	0.88	0.80	0.96
Asian Indian, immigrant	5.77	0.11	0.59	0.57	0.62	13.61	0.15	1.83	1.77	1.88
Chinese, US-born	5.49	0.20	0.59	0.55	0.64	9.93	0.24	0.77	0.72	0.81
Chinese, immigrant	7.33	0.11	0.41	0.39	0.42	16.40	0.14	1.72	1.68	1.76
Filipino, US-born	6.52	0.23	0.79	0.73	0.85	12.66	0.29	0.94	0.88	0.99
Filipino, immigrant	9.94	0.14	0.77	0.74	0.79	11.74	0.14	1.53	1.48	1.58
Japanese, US-born	14.77	0.33	0.87	0.83	0.92	5.24	0.18	0.68	0.62	0.74
Japanese, immigrant	7.51	0.30	0.41	0.38	0.44	10.02	0.30	1.25	1.15	1.35
Korean, US-born	5.20	0.34	0.61	0.54	0.69	20.11	0.55	1.50	1.38	1.62
Korean, immigrant	6.80	0.16	0.41	0.39	0.44	28.06	0.27	4.06	3.94	4.20
Vietnamese, US-born	5.29	0.36	0.70	0.61	0.80	25.18	0.67	1.58	1.46	1.70
Vietnamese, immigrant	10.25	0.19	0.57	0.54	0.59	22.34	0.24	1.76	1.70	1.82
Cambodian, US-born	5.43	0.76	0.69	0.51	0.92	35.22	1.56	1.44	1.24	1.67
Cambodian, immigrant	15.29	0.63	0.73	0.65	0.81	22.39	0.70	1.31	1.20	1.44
Bangladeshi, US-born	4.74	2.17	0.53	0.20	1.38	14.77	3.91	1.10	0.65	1.87
Bangladeshi, immigrant	6.74	0.62	0.53	0.43	0.66	28.14	1.08	2.37	2.10	2.67
Pakistani, US-born	5.04	0.79	0.64	0.43	0.95	22.23	1.62	1.59	1.30	1.94
Pakistani, immigrant	6.99	0.36	0.57	0.51	0.64	30.29	0.62	3.26	3.04	3.50
Hmong, US-born	4.28	0.65	0.59	0.43	0.82	26.57	1.44	1.15	0.99	1.34
Hmong, immigrant	14.37	0.83	0.74	0.64	0.85	18.40	0.88	0.81	0.71	0.92
Laotian, US-born	4.97	0.90	0.71	0.51	1.00	33.07	1.80	1.53	1.29	1.82
Laotian, immigrant	12.33	0.66	0.69	0.61	0.78	17.67	0.72	1.05	0.94	1.17
Thai, US-born	3.45	0.85	0.56	0.35	0.90	22.97	1.84	1.53	1.22	1.94
Thai, immigrant	6.77	0.46	0.43	0.37	0.50	24.13	0.72	2.48	2.26	2.73
Other Asians, US-born	5.98	0.38	0.68	0.59	0.77	13.49	0.51	0.83	0.75	0.92
Other Asians, immigrant	7.04	0.24	0.53	0.49	0.57	21.04	0.36	1.73	1.64	1.82
Native Hawaiian	15.17	0.65	1.06	0.96	1.17	13.90	0.59	0.88	0.79	0.98
Samoan	13.49	0.91	1.04	0.88	1.23	21.38	1.05	0.98	0.86	1.13
Guamanian	11.02	0.92	0.99	0.82	1.19	19.23	1.04	1.08	0.92	1.27
Other Pacific Islanders, US-born	8.44	1.27	0.79	0.57	1.08	24.61	1.87	1.22	0.98	1.51
Other Pacific Islanders, immigrant	12.61	1.29	0.64	0.49	0.83	25.01	1.62	1.70	1.38	2.10
All other groups, US-born	18.89	0.14	1.72	1.69	1.76	20.32	0.13	1.20	1.17	1.22
All other groups, immigrant	9.93	0.25	0.72	0.68	0.77	24.59	0.34	2.11	2.02	2.21

OR: odds ratio; SE: standard error; CI: confidence interval.

^1^Adjusted by logistic regression model for age, gender, marital status, education, poverty, and employment status.

**Table 6:** Prevalence and adjusted odds of lack of health insurance among people with disabilities according to race/ethnicity: The 2008–2010 American Community Survey (*N* = 1,233,595).

Race/ethnicity	All ages					Age ≥ 18 years				
	Prevalence		Adjusted odds ratio^1^			Prevalence		Adjusted odds ratio^2^		
	%	SE	OR	95% CI		%	SE	OR	95% CI	
Non-Hispanic White	8.22	0.03	1.00	Reference		8.45	0.03	1.00	Reference	
Mexican	22.69	0.16	1.70	1.66	1.75	24.69	0.17	1.64	1.60	1.69
Puerto Rican	10.14	0.24	0.78	0.73	0.82	11.32	0.27	0.76	0.72	0.81
Cuban	12.61	0.39	1.16	1.06	1.27	12.90	0.41	1.17	1.06	1.28
Central/South American	21.98	0.34	1.42	1.35	1.50	23.62	0.37	1.33	1.26	1.40
Other Hispanics	12.75	0.33	1.11	1.04	1.20	13.80	0.36	1.11	1.03	1.19
Non-Hispanic Black	13.11	0.08	1.07	1.05	1.09	14.01	0.09	1.05	1.03	1.07
American Indian/AN	20.01	0.34	1.84	1.75	1.93	20.84	0.36	1.84	1.75	1.94
Asian Indian	13.14	0.57	1.23	1.09	1.38	13.84	0.61	1.39	1.24	1.57
Chinese	7.56	0.32	0.78	0.69	0.87	7.83	0.34	0.83	0.74	0.94
Filipino	7.81	0.34	0.80	0.72	0.89	7.95	0.35	0.86	0.77	0.97
Japanese	2.28	0.26	0.47	0.35	0.63	2.26	0.26	0.50	0.37	0.66
Korean	13.31	0.67	1.12	0.96	1.29	13.73	0.70	1.23	1.06	1.42
Vietnamese	10.47	0.51	0.63	0.56	0.72	10.59	0.53	0.65	0.57	0.73
Cambodian	13.97	1.33	0.63	0.49	0.82	14.64	1.44	0.63	0.49	0.82
Bangladeshi	20.09	3.37	0.98	0.60	1.60	20.92	3.68	1.05	0.63	1.76
Pakistani	25.13	1.98	1.60	1.23	2.08	27.30	2.17	1.98	1.51	2.59
Hmong	10.43	1.59	0.35	0.24	0.51	10.06	1.68	0.36	0.25	0.53
Laotian	13.45	1.60	0.65	0.48	0.88	14.41	1.71	0.65	0.48	0.89
Thai	8.95	1.62	0.46	0.28	0.74	9.47	1.72	0.47	0.29	0.77
Other Asians	12.45	0.92	0.80	0.66	0.96	13.40	1.01	0.80	0.66	0.96
Native Hawaiian	9.96	1.13	0.91	0.67	1.24	10.55	1.20	0.86	0.62	1.17
Samoan	14.36	2.48	1.12	0.76	1.64	14.79	2.61	1.08	0.72	1.63
Guamanian	8.82	2.37	1.00	0.58	1.73	8.99	2.46	0.84	0.47	1.48
Other Pacific Islanders	25.86	3.50	1.47	0.92	2.35	25.94	3.64	1.33	0.82	2.16
All other groups	14.29	0.22	1.19	1.14	1.24	16.61	0.26	1.20	1.15	1.25

^1^Adjusted by logistic regression model for age, gender, immigrant status, and poverty level.

^2^Adjusted by logistic regression model for age, gender, immigrant status, marital status, education, poverty, and employment status.
